# Study on the preventive effect of Neiguan acupoint stimulation combined with Zusanli acupoint stimulation on postoperative nausea and vomiting in patients undergoing hysteroscopic surgery

**DOI:** 10.1186/s12906-026-05451-x

**Published:** 2026-06-26

**Authors:** Hongliang Gao, Zhifang Li, Wei Hao, Xiaoguang Liu, Mengmeng Liang, Li Yang, Yan Li, Yajing Dong, Chunlei Zhang

**Affiliations:** 1 Department of Anesthesiology, Hebei Provincial Hospital of Traditional Chinese Medicine, No. 389, Zhongshan East Road, Chang’an District, Shijiazhuang, 050000 Hebei China; 2 Department of Cardiology, Fourth People’s Hospital of Hengshui City, Hengshui, Hebei 053000 China; 3 Department of Physical Examination, the Eighth People’s Hospital of Hebei Province, Shijiazhuang, 050022 Hebei China

**Keywords:** Neiguan acupoint, Zusanli, Hysteroscope, Nausea and vomiting

## Abstract

**Objective:**

To explore the preventive effect of thumbtack needle Neiguan acupoint stimulation combined with Zusanli acupoint stimulation on postoperative nausea and vomiting in patients undergoing hysteroscopic surgery. Experimental procedure: Using convenient sampling, 90 patients scheduled for hysteroscopic surgery were enrolled and randomly assigned to group A (Neiguan acupoint stimulation combined with Zusanli acupoint stimulation group), group B (Neiguan acupoint stimulation group), and group C (Streitberger sham acupuncture group). Postoperative nausea, vomiting and pain indicators were collected for comparison.

**Results:**

The incidence of postoperative nausea in group A and group B was lower than that in group C at 0 ~ 6 h, and the incidence of postoperative vomiting in group A and group B was lower than that in group C at 0 ~ 6 h and 6 ~ 12 h. There were significant differences in VAS scores and postoperative exhaust time between the three groups at 6,12 and 24 h after operation. Further pairwise comparison showed that the VAS scores and postoperative exhaust time of group A at 6,12 and 24 h after operation were the smallest (*P* < 0.05).

**Conclusion:**

Combined acupoint stimulation at Zusanli and Neiguan was more effective in reducing the severity of nausea and vomiting and accelerating postoperative recovery than stimulation of Neiguan alone or sham control.

## Introduction

Hysteroscopy enters the uterine cavity by using the anterior part of the mirror body, and then visually and clearly observes the size, location, appearance, range and tissue structure of the lesion surface [1]. It is of great significance for the diagnosis and treatment of intrauterine adhesions, abnormal uterine bleeding, intrauterine device placement, removal, infertility and other intrauterine diseases and gynecological hemorrhagic diseases. Because of the need to expand, stretch and expand the uterine cavity during hysteroscopy, patients are prone to cervical injury, bleeding and other symptoms, accompanied by pain and discomfort. Patients with obvious pain are prone to unconscious body movement and cannot cooperate with hysteroscopy. In severe cases, they even have symptoms such as arrhythmia, blood pressure drop, palpitation and chest tightness, resulting in extreme instability of hemodynamics, and may also cause complications such as uterine perforation, hyponatremia and venous air embolism [2], which is not conducive to the smooth progress of hysteroscopy. In recent years, with the continuous development of social economy and medical level, comfortable diagnosis and treatment has become a new trend of current medical treatment. Painless hysteroscopy refers to the use of a certain amount of analgesic and sedative drugs for hysteroscopy, which is helpful to improve the comfort of patients and shorten the examination time. It is widely used in the diagnosis and treatment of gynecological diseases [3–4]. Therefore, in the process of hysteroscopy diagnosis and treatment, it is of great significance to adopt perfect and appropriate analgesic and sedative drugs to improve the comfort of patients and improve the safety of hysteroscopy. Due to the short operation time of hysteroscopy, patients need to recover quickly after operation, and the operation requirements of anesthesia are high. It not only needs patients to stay awake after discharge, but also needs no anesthetic drug residues and sequelae [5]. At present, the anesthetic drugs for painless hysteroscopy include simple use of sedative drugs such as dexmedetomidine, propofol, etomidate, and compound benzodiazepines, opioid analgesics and other drugs.

Postoperative nausea and vomiting (PONV) is a common surgical complication [6]. About 30% of surgical patients have experienced PONV, and this proportion can even reach 80% in high-risk patients [6–7]. In order to avoid PONV as much as possible, patients are even willing to pay an additional $ 100–200 in medical expenses, which will greatly increase medical costs [8–10]. Although PONV itself is not fatal, it may lead to wound dehiscence and incisional hernia formation. Repeated nausea and vomiting can lead to dehydration, electrolyte imbalance, and even asphyxia due to aspiration of vomit into the lung [8]. The main existing preventive drugs include antihistamines (H1RA), serotonin receptor blockers (5HTRA), dopamine receptor blockers (D2RA) and glucocorticoids. Among them, 5HTRA is the first-line drug for the prevention and treatment of PONV [9]. However, there are still many adverse reactions in the drug, which limits its clinical promotion and use.

Nausea and vomiting are one of the dominant diseases of acupuncture treatment. Although the mechanism of acupuncture treatment of nausea and vomiting is not fully understood [10], studies have shown that acupuncture therapy can reduce the incidence of nausea and vomiting after surgery, reduce the severity of nausea after surgery, reduce the frequency and overall dose of anti-vomiting drugs after surgery, and has the advantages of improving patients’ satisfaction with clinical treatment and reducing patients ' economic burden [11]. Neiguan (PC6) is one of the effective acupoints for the prevention of PONV, which has been proved to be one of the effective acupoints for the prevention of PONV. It is located on the palm side of the forearm, 2 inches above the wrist transverse line, between the palmaris longus tendon and the radial flexor tendon. A Cochrane study compared PC6 acupoint stimulation with six different antiemetic drugs (metoclopramide, benzoxazine, prochloraz, droperidol, ondansetron and dexamethasone) and found that there was no difference between PC6 acupoint stimulation and the above standard antiemetic drugs in preventing PONV [12]. PC6 and Zusanli (ST36) are often stimulated at the same time, resulting in a synergistic effect, promoting the recovery of gastrointestinal function and improving patient satisfaction [13–14]. At present, the effect of PC6 combined with ST36 on preventing nausea and vomiting after hysteroscopic surgery has not been discussed. Therefore, this study aimed to compare the preventive effects on PONV following hysteroscopic surgery among three interventions: combined PC6 and ST36 stimulation, PC6 stimulation alone, and a sham control, in order to evaluate whether the combination offers a superior clinical benefit.

## Study subjects and methods

### Study subjects

**Fig. 1:**
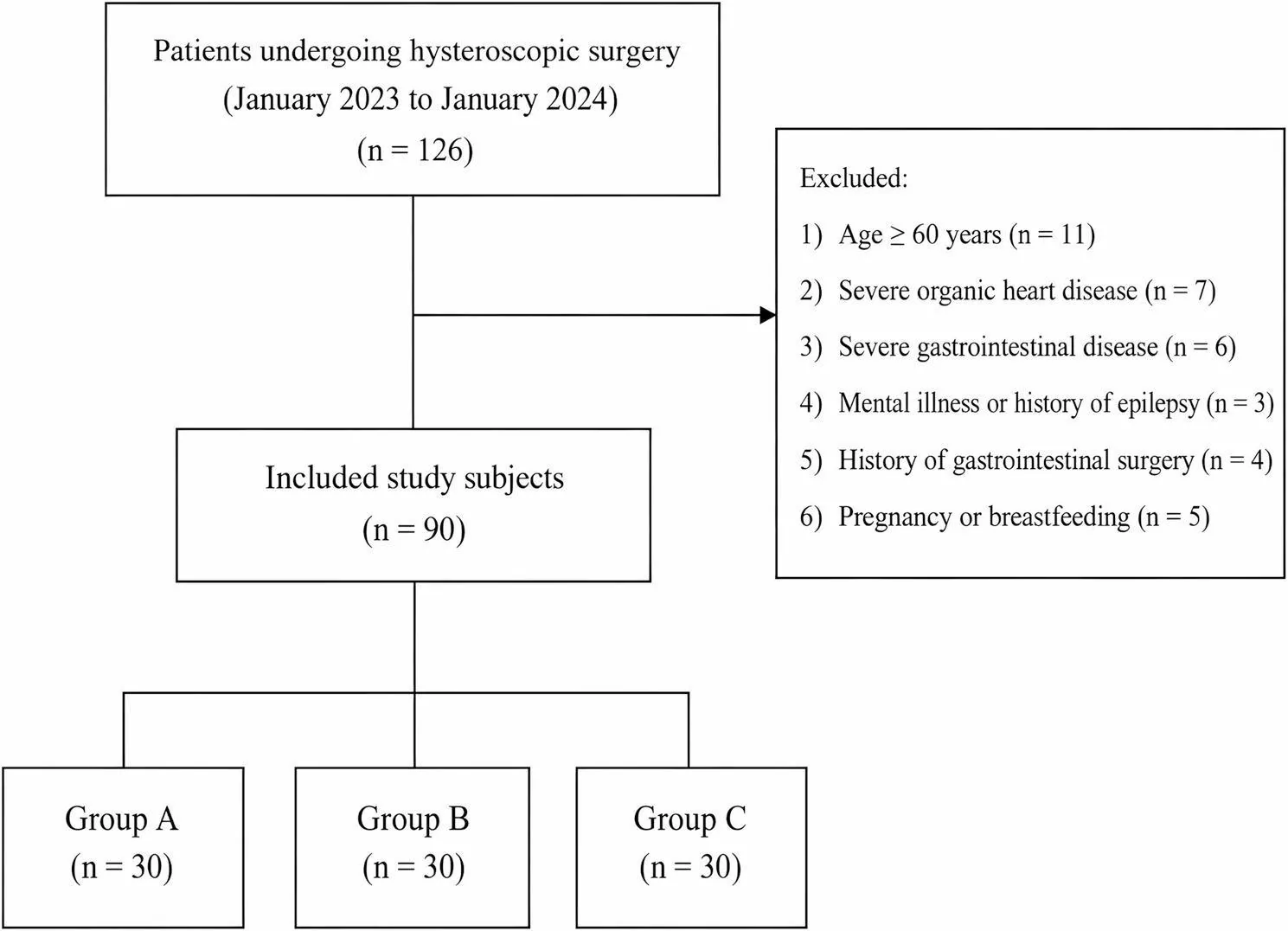
The flowchart of the research. Group A, Neiguan acupoint stimulation combined with Zusanli acupoint stimulation group; Group B, Neiguan acupoint stimulation alone group; Group C, Streitberger sham acupuncture group

In this study, 90 patients who underwent hysteroscopic surgery from January 2023 to January 2024 were randomly assigned to three groups using a computer-generated random sequence: group A (Neiguan acupoint stimulation combined with Zusanli acupoint stimulation group), group B (Neiguan acupoint stimulation group), and group C (Streitberger sham acupuncture group), with 30 cases in each group (Fig. 1). The allocation was concealed using sealed envelopes, and grouping was performed by an independent statistician. This study adopted a patient- and assessor-blind design. Due to the nature of the interventions, the acupuncturists performing the procedures could not be blinded to the group assignment. However, the patients and the outcome assessors (the nursing staff who collected postoperative data) were blinded to the group allocations throughout the trial. To maintain blinding, patients in all three groups underwent an identical skin disinfection procedure and had devices (real or sham) applied by the same trained physicians. The sham acupuncture device was designed to visually mimic the real thumbtack needle and produced a similar tactile sensation upon skin contact without piercing the skin. Patients were not informed about the specific acupuncture sensation (‘deqi’) they might experience and were given standardized instructions prior to the intervention.

Criteria for inclusion: (1) 18–60 years old; (2) American Society of Anesthesiologists (ASA) classification: I ~ II; (3) Patients who can tolerate anesthesia and hysteroscopy. Exclusion criteria: (1) severe organic heart disease; (2) severe gastrointestinal disease; (3) mental illness or history of epilepsy; (4) Antiemetic drugs, anti-acid drugs or nausea and vomiting were taken within 24 h before operation; (5) History of gastrointestinal surgery; (6) Pregnancy or breastfeeding.

Separation or removal criteria: (1) Patients who experience water, electrolyte disorders causing nausea or vomiting after surgery, or who experience serious complications after surgery; (2) In the course of the study, due to fainting, intolerance to acupuncture or serious adverse events; (3) Subjects who were unable to cooperate with the completion of all treatments and examinations due to poor compliance or other factors during the study; (4) The combined application of drugs that affect the acupuncture effect within the non-specified range affects the judgment of efficacy and safety.

This study was approved by the Ethics Committee of Hebei Provincial Hospital of Traditional Chinese Medicine [HBZY2020-KY-054-02]. All patients provided written informed consent, and the study was registered in the Chinese Clinical Trial Registry (ChiCTR2200067239, https://www.chictr.org.cn/index.html). There were no deviations or changes from the registered protocol during the conduct of the trial.

Sample size calculation: The sample size for this study was determined a priori based on the primary outcome of PONV incidence within the first 24 h. Based on recent randomized controlled trials, the anticipated incidence of PONV in patients undergoing hysteroscopic surgery with conventional antiemetic prophylaxis (similar to our control group) ranges from 19.9% to 38.8% [15, 16]. We hypothesized that combined acupoint stimulation (Group A) would reduce the PONV incidence to approximately 10%. Using PASS 15.0 software (NCSS, LLC), with a two-sided alpha level of 0.05 and a statistical power (1-β) of 80%, a sample size of 66 patients (22 per group) was calculated to detect this effect size. To account for a potential 10% attrition rate and to balance the group sizes for repeated measures ANOVA, we increased the total sample size to 90 (30 patients per group).

### Study methods

All patients were treated with standardized anesthesia. After preparation, anesthesia induction was performed in a rapid sequence: midazolam 0.05 mg/kg, sufentanil 0.5 µg/kg, etomidate 0.2 mg/kg, and cis-atracurium 0.15 mg/kg. Before the patient’s consciousness disappeared, the patient was instructed to breathe spontaneously, and the oxygen flow was increased to 4–5 L/min. When the patient’s consciousness disappeared, the mask was gently buckled. After the patient’s breathing disappeared, the jaw was lifted, the mask was pressurized for 3 min, and then 3 # laryngeal mask was placed. Connect the anesthesia machine to determine the position of the fixed laryngeal mask after effective ventilation. Volume-controlled ventilation, tidal volume range of 6-8 ml/kg. The end-tidal carbon dioxide monitoring device was connected to the end-tidal carbon dioxide monitoring device, and the respiratory frequency was adjusted to make the value between 35 and 45 mmHg. Anesthesia was maintained with intravenous infusion of propofol and remifentanil, the doses of which were titrated to maintain the Bispectral Index (BIS) value within a range of 40–60. Cisatracurium was added intermittently according to the needs of the operation. Within 20–30 min before the end of the operation, granisetron 3 mg and dezocine 10 mg were given to prevent vomiting and analgesia. After the operation, the infusion of anesthesia maintenance drugs was stopped, and the disposable intravenous analgesia pump was connected. The drugs were sufentanil and butorphanol, with a total volume of 100 ml. The surgical towel and monitoring equipment were removed, and the patient was quickly transferred to the post-anesthesia care unit (PACU) and connected to the anesthesia machine. The respiratory parameters were the parameters of the anesthesia machine when the patient left the room. Waiting for the patient to wake up naturally, the anesthesiologist in the recovery room evaluated the indication of removing the laryngeal mask. After a certain period of monitoring, the patient was transferred to the general ward and handed over to the ward physician and nurse to confirm that it was correct.

Acupuncture method: The patient’s knees were bent, and the palms of the patient ‘s hands were brought together. The horizontal stripe of the middle finger was three inches, and it was placed under the depression below the outside of the patella, which was the Zusanli point. Neiguan acupoint selection: Between the palmaris longus tendon and the flexor carpi radialis, 2 inches on the wrist stripes, about three transverse fingers of the site to take points. Acupuncture process: Thirty minutes before the operation, the anesthesiology nurses disinfected the skin of Zusanli and Neiguan points with alcohol, and put the needle (1.5 mm × 2.0 mm) into the bilateral Zusanli and Neiguan points quickly and vertically by wearing sterile gloves. The position refers to the ' meridian point ' (GB12346-90), and press the needle cap to make the patient have a sense of soreness or numbness, but no obvious pain, can or can not achieve ' deqi ‘. The patient pressed the needle cap again before leaving the operating room, and updated the needle once 24 hours after the operation. If the needle fell off, wet, and oozed blood, it should be replaced in time. Once nausea, vomiting, abdominal distension and other symptoms occurred in the thumbtack needle group, the patients or their families pressed the thumbtack needle cap to ' deqi ' by themselves, and the course of treatment was 3 days.

**Fig. 2:**
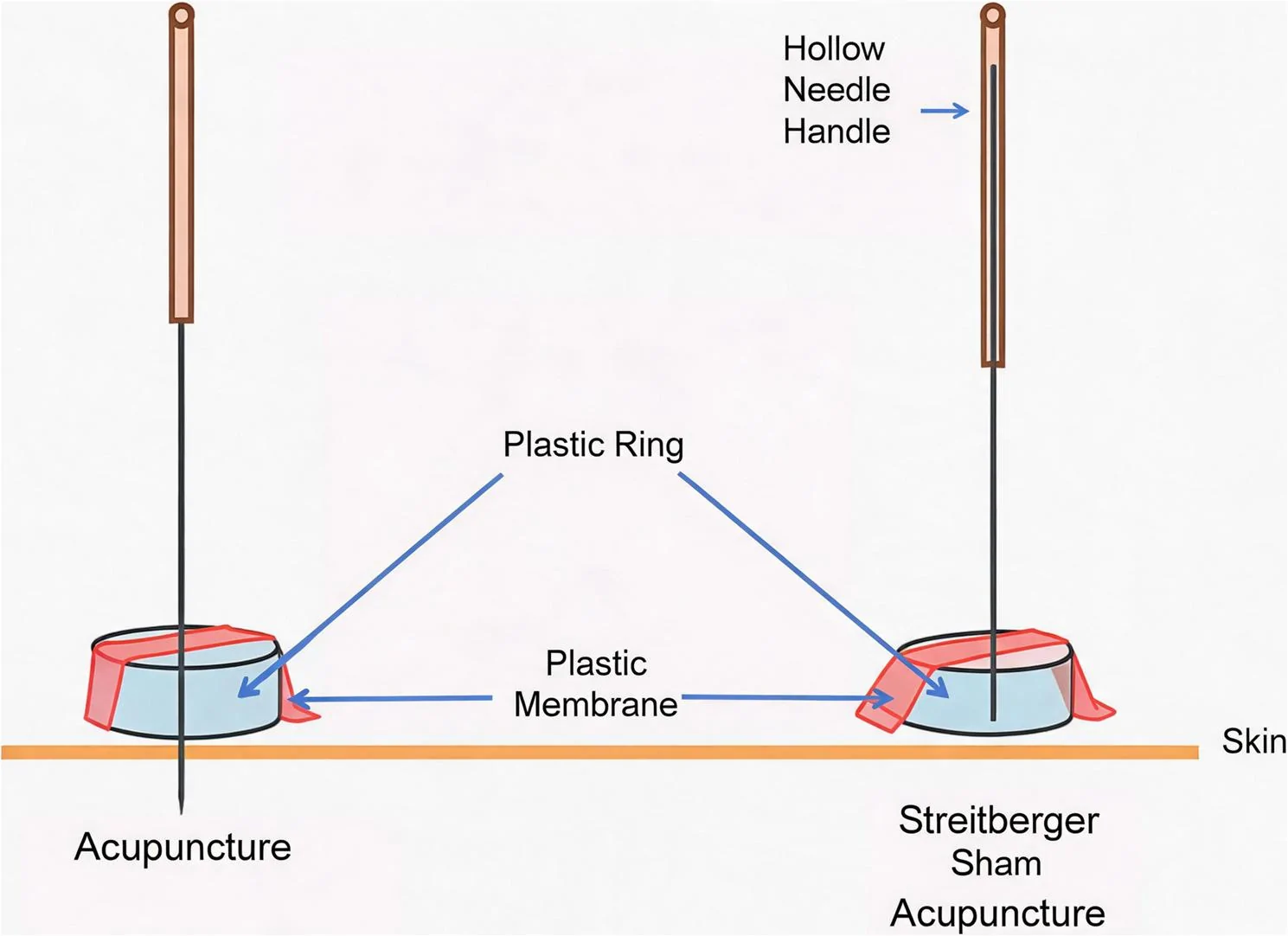
Schematic diagram of the verum and sham acupuncture needles. The left panel shows a conventional acupuncture needle penetrating the skin. The right panel shows the Streitberger sham acupuncture device used in the sham control group. The blunt-tipped needle retracts into a hollow handle upon contact with the skin, producing a tactile sensation similar to needle insertion without actual skin penetration

Streitberger sham acupuncture method: The Streitberger placebo acupuncture device (Asiamed GmbH, Grassau, Germany; based on Patent No. DE102007053564) was used. This special blunt needle device makes surface contact at the standard acupoint locations (PC6/ST36). When the needle body contacts the skin, the spring recoile mechanism is used to simulate the real acupuncture feeling without piercing the skin, and the operation is maintained at 90° vertical Angle and 50–100 g contact pressure for 30 s. All procedures are performed by specially trained physicians, using the same appearance and operating environment as the real acupuncture group to ensure the effectiveness of the blind method (Fig. 2).

To ensure the integrity of blinding, a subset of 30 patients (10 randomly selected from each group) was interviewed upon completion of the 24-hour follow-up using a patient blinding questionnaire. They were asked which treatment they believed they had received: ‘real acupuncture’, ‘sham acupuncture’, or ‘do not know’. The success of blinding was initially assessed by comparing the distribution of patients’ treatment guesses across groups using the Chi-square test. In addition, the Bang blinding index was calculated for each group to provide a quantitative assessment of blinding success. A blinding index close to 0 was interpreted as indicating a response pattern compatible with random guessing, whereas values approaching 1 suggested correct identification of the assigned intervention and values approaching − 1 suggested opposite guessing. Importantly, the Streitberger sham needle, by design, does not puncture the skin and therefore does not elicit a genuine ‘deqi’ sensation. However, the spring-loaded mechanism creates a tactile cue and a mild sensation upon contact, which, combined with the visual setup and the authoritative context of a clinical procedure, helped to maintain the patient’s belief in the treatment allocation.

### Quality control

In order to prevent the differences in skills caused by the clinical work background and acupuncture education background of doctors, high-qualified acupuncture doctors with more than 10 years of employment were selected for acupuncture therapy. The researchers fill in each selected case, complete data collection, and data entry and management.

### Statistical analysis

Statistical analysis was performed using SPSS 26.0 statistical software. The normality test was performed using the K-S method. The measurement data that satisfied the normality were expressed as mean ± standard deviation (¯x ± s). The mean comparison between multiple groups was performed using one-way analysis of variance, and the pairwise comparison was performed using the SNK method. Repeated measures analysis of variance was used for repeated measurement data. The count data were expressed as frequency (n) or rate (%). The χ^2^ test was used for those who met the conditions, and the Fisher exact probability method was used for those who did not meet the conditions. Two-sided *P* < 0.05 was considered statistically significant.

## Results

### General information

The results showed that there were 30 cases in group A, with an average age of 42.33 ± 7.73 years and an average BMI of 23.05 ± 1.51 kg/m^2^. There were 30 patients in group B, with an average age of 41.10 ± 11.02 years and an average BMI of 23.86 ± 1.61 kg/m^2^. There were 30 patients in group C, with an average age of 39.87 ± 7.51 years and an average BMI of 22.95 ± 2.17 kg/m^2^. There was no significant difference in age, BMI, motion sickness history and smoking history among the three groups (*P* > 0.05), see Table 1.

**Table 1:** General information

Item	Group A (*n* = 30)	Group B (*n* = 30)	Group C (*n* = 30)	*P* value
Age (years old)	42.33 ± 7.73	41.10 ± 11.02	39.87 ± 7.51	0.564
BMI (kg/m2)	23.05 ± 1.51	23.86 ± 1.61	22.95 ± 2.17	0.105
Postoperative use of opioids (case)	28	29	30	0.770^*^
History of motion sickness (case)	14	18	17	0.644^*^
Smoking history (case)	2	1	0	
ASA grade (case)				1.000
Grade I	6	7	6	
Grade II	24	23	24	
Hypertension (case)	3	2	3	1.000^*^
Diabetes (case)	3	2	1	0.868^*^
Coronary heart disease (case)	2	2	2	1.000^*^

Note: Group A, Neiguan acupoint stimulation combined with Zusanli acupoint stimulation group; Group B, Neiguan acupoint stimulation alone group; Group C, Streitberger sham acupuncture group. Data are presented as mean ± standard deviation or number of patients, as appropriate

*BMI* Body mass index, *ASA* American Society of Anesthesiologists

^*^Fisher’s exact probability test was used

### Surgical data comparison

The results showed that there was no significant difference in operation time, anesthesia time and intraoperative fluid volume among the three groups (*P* > 0.05). There were significant differences in sufentanil dosage, T2 mean arterial pressure and anesthesia recovery time among the three groups (*P* < 0.05), as shown in Table 2.

**Table 2:** Comparison of surgical data

Item	Group A (*n* = 30)	Group B (*n* = 30)	Group C (*n* = 30)	*P* value
Operation time (minutes)	84.27 ± 11.05	84.00 ± 9.75	87.03 ± 10.88	0.472
Anesthesia time (minutes)	102.73 ± 9.57	99.03 ± 13.33	101.50 ± 13.88	0.504
Intraoperative fluid Infusion volume (mL)	805.03 ± 122.37	794.13 ± 129.63	806.30 ± 144.05	0.926
Anesthetic dosage				
Sufentanil (µg)	22.28 ± 1.62	21.38 ± 3.37	24.54 ± 3.62	< 0.001
Propofol (mg)	510.27 ± 24.46	513.50 ± 24.61	505.90 ± 30.68	0.545
Remifentanil (mg)	1.34 ± 0.16	1.28 ± 0.37	1.20 ± 0.09	0.084
Mean arterial pressure at T1 (mmHg)	91.57 ± 4.90	89.73 ± 7.80	89.07 ± 10.19	0.453
Mean arterial pressure at T2 (mmHg)	83.10 ± 7.08	82.17 ± 4.71	76.47 ± 6.71	< 0.001
Mean arterial pressure at T3 (mmHg)	91.07 ± 6.83	90.83 ± 6.95	92.43 ± 4.72	0.566
T1 heart rate (times/minute)	84.93 ± 9.67	84.50 ± 7.75	81.17 ± 14.88	0.365
T2 heart rate (times/minute)	69.70 ± 7.89	71.97 ± 5.46	69.93 ± 7.51	0.394
T3 heart rate (times/minute)	85.43 ± 8.86	86.93 ± 6.97	84.83 ± 5.75	0.521
Anesthesia recovery time (minutes)	21.53 ± 2.53	19.17 ± 3.53	19.87 ± 3.14	0.012

Note: Group A, Neiguan acupoint stimulation combined with Zusanli acupoint stimulation group; Group B, Neiguan acupoint stimulation alone group; Group C, Streitberger sham acupuncture group. Data are presented as mean ± standard deviation. T1, before anesthesia induction; T2, during surgery; T3, at the end of surgery

### Contrasting follow-up data

Among the 30 randomly selected patients who completed the post-intervention blinding questionnaire, there was no significant difference in the distribution of treatment guesses among the three groups (*P* > 0.05). The Bang blinding index was close to 0 in each group, indicating that patients were unable to reliably identify their assigned intervention and that patient blinding was generally maintained.

The results showed that there were 2 cases of nausea and 1 case of vomiting in group A at 0 ~ 6 h, 2 cases of nausea and 0 case of vomiting at 6 ~ 12 h, 1 case of nausea and 0 case of vomiting at 12 ~ 24 h. In group B, there were 5 cases of nausea and 4 cases of vomiting in 0 ~ 6 h, 5 cases of nausea and 3 cases of vomiting in 6 ~ 12 h, 2 cases of nausea and 2 cases of vomiting in 12 ~ 24 h. In group C, there were 13 cases of nausea and 9 cases of vomiting in 0 ~ 6 h, 7 cases of nausea and 8 cases of vomiting in 6 ~ 12 h, 1 case of nausea and 1 case of vomiting in 12 ~ 24 h. The incidence of postoperative nausea in group A and group B was lower than that in group C at 0 ~ 6 h, and the incidence of postoperative vomiting in group A and group B was lower than that in group C at 0 ~ 6 h and 6 ~ 12 h.

**Fig. 3:**
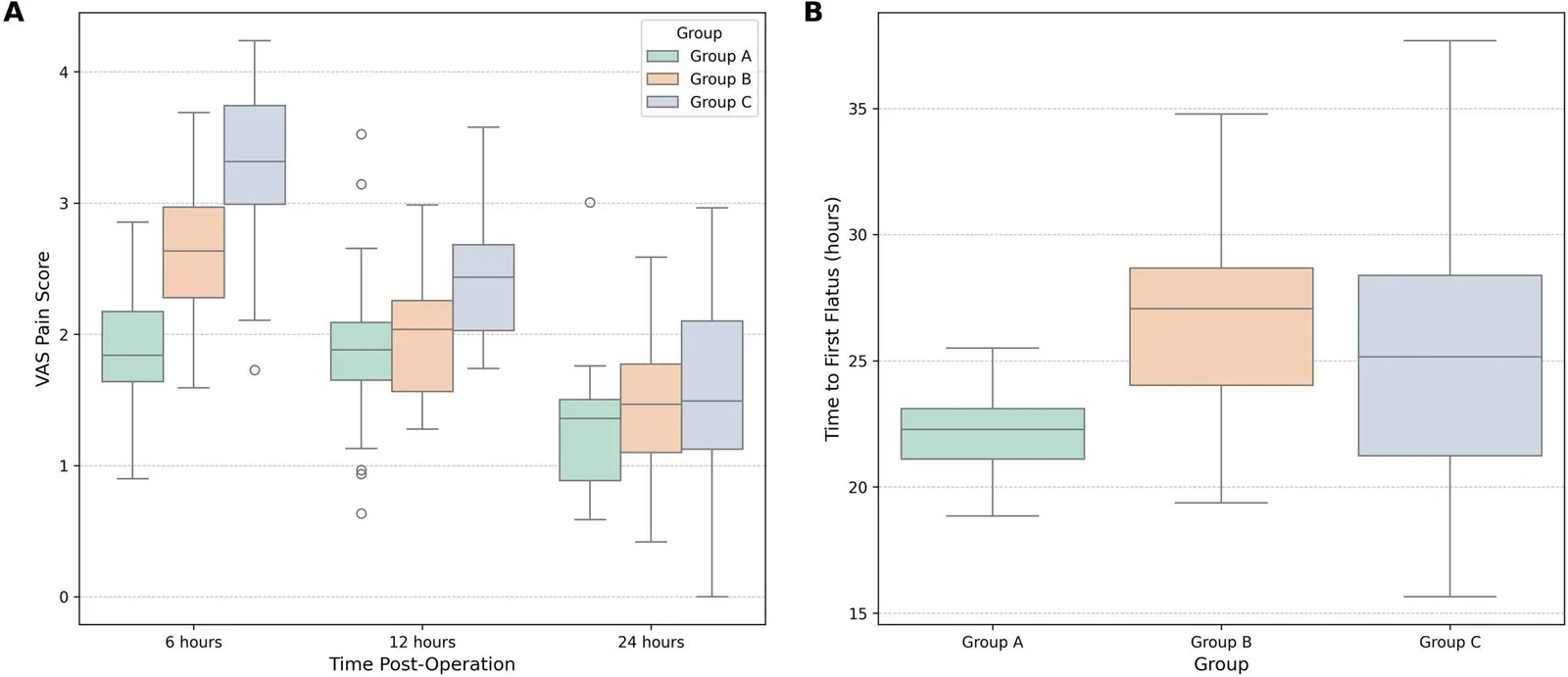
Distribution of primary and key secondary outcomes. Group A, Neiguan acupoint stimulation combined with Zusanli acupoint stimulation group; Group B, Neiguan acupoint stimulation alone group; Group C, Streitberger sham acupuncture group. Panel **A** shows VAS pain scores at 6, 12, and 24 hours after surgery. Panel **B** shows the time to first postoperative flatus. In each box plot, the central line indicates the median, the box edges represent the interquartile range, and the whiskers extend to the most extreme data points within 1.5 times the interquartile range

There were significant differences in VAS scores and postoperative exhaust time between the three groups at 6,12 and 24 h after operation. Further pairwise comparison showed that the VAS scores and postoperative exhaust time of group A at 6,12 and 24 h after operation were the smallest (*P* < 0.05) (Table 3, Fig. 3).

**Table 3:** Comparison of postoperative follow-up outcomes among the three groups

Item	Time	Group A (*n* = 30)	Group B (*n* = 30)	Group C (*n* = 30)	*P* value
Postoperative nausea, n (%)	0–6 h	2 (6.7)	5 (16.7)	13 (43.3)	0.002
	6–12 h	2 (6.7)	5 (16.7)	7 (23.3)	0.238^*^
	12–24 h	1 (3.3)	2 (6.7)	1 (3.3)	1.000^*^
Postoperative vomiting, n (%)	0–6 h	1 (3.3)	4 (13.3)	9 (30.0)	0.018^*^
	6–12 h	0 (0.0)	3 (10.0)	8 (26.7)	0.005^*^
	12–24 h	0 (0.0)	2 (6.7)	1 (3.3)	0.770^*^
Postoperative VAS score	Postoperative 6 h	1.97 ± 0.56^#&^	2.67 ± 0.55	3.30 ± 0.60	< 0.001
	Postoperative 12 h	1.90 ± 0.66^#&^	2.00 ± 0.45	2.30 ± 0.47	0.014
	Postoperative 24 h	1.27 ± 0.45^#&^	1.43 ± 0.50	1.67 ± 0.61	0.014
Time to first postoperative flatus, hours	—	22.03 ± 1.63^#&^	25.03 ± 4.66	26.57 ± 7.54	0.004

Note: Group A, Neiguan acupoint stimulation combined with Zusanli acupoint stimulation group; Group B, Neiguan acupoint stimulation alone group; Group C, Streitberger sham acupuncture group. For postoperative nausea and postoperative vomiting, data are presented as the number and percentage of patients experiencing the symptom during each postoperative time interval, n (%)

Continuous variables are presented as mean ± standard deviation

*VAS* Visual analog scale

^*^Fisher’s exact probability test was used

^#^Compared with Group C, the difference was statistically significant

^&^Compared with Group B, the difference was statistically significant

## Discussion

Nausea and vomiting are one of the dominant diseases of acupuncture treatment. Although the mechanism of acupuncture treatment of nausea and vomiting is not fully understood [10], studies have shown that acupuncture therapy can reduce the incidence of nausea and vomiting after surgery, reduce the severity of nausea after surgery, reduce the frequency and overall dose of anti-vomiting drugs after surgery, and has the advantages of improving patients’ satisfaction with clinical treatment and reducing patients’ economic burden [11]. The results of this study show that intradermal needle Neiguan acupoint stimulation combined with Zusanli acupoint stimulation can more effectively reduce the occurrence of hysteroscopic PONV than conventional antiemetic measures and single acupoint stimulation. Thumb-tack needle therapy has been recorded in ancient books, which belongs to superficial needling or superficial needling in traditional acupuncture methods. There is no need to immediately produce a sense of ' deqi ' when applying the needle, and after embedding the needle, it can ' wait for qi ' and ' regulate qi ‘, which is the specific application of the combination of skin theory and acupoint theory [17]. After the needle is applied, it will produce a lasting and stable stimulation to the body. With the extension of the needle retention time and the intermittent pressing of the acupuncture site, it gradually produces a sense of soreness and distention, which is similar to the ordinary needle sensation and has the effect of treating the disease [18]. Thumb-tack needle is easy to operate, almost no needle sense when applying needles, high safety, and does not affect patient activities. Therefore, this study selected thumb-tack needle as a needle for acupuncture and moxibustion.

At present, a large number of clinical studies have shown that [19–21], acupuncture at Neiguan has a significant effect on PONY, which can effectively reduce the frequency and time of PONY attack. Shi et al. [19] systematically evaluated the exact effect of Neiguan point in the treatment of nausea and vomiting after pediatric tonsillectomy, and proved that the use of Neiguan point in the treatment of pediatric tonsil POVN is economical and effective, and the risk rate is lower than that of conventional treatment. Studies have shown that [22], although Neiguan point is often combined with Zusanli, Gongsun, Hegu and other points to further play the role of antiemetic and antiemetic, but single stimulation of Neiguan point can effectively play the role of prevention and treatment of PONV. According to the existing research, stimulating Neiguan acupoint to prevent and treat postoperative nausea and vomiting mainly plays the specific role of Neiguan acupoint, but its mechanism is not clear. Zhu et al. [23] analyzed the mechanism of stimulating Neiguan point to prevent and treat postoperative nausea and vomiting by sorting out the literature, and believed that stimulating Neiguan point could mediate the release of β-endorphin in cerebrospinal fluid and stimulate endogenous µ receptor to exert antiemetic effect. It may also be related to changes in serotonin release. Stoicea N et al. [24] thought that acupuncture or electroacupuncture stimulation of Neiguan acupoint can effectively prevent PONV and exert analgesic effect, mainly through the release of opioid peptides and the regulation of 5-hydroxytryptamine, norepinephrine, tachykinins and endogenous endorphin system. However, the effect of high-frequency and low-frequency electroacupuncture is different. Low-frequency stimulation of opioid neuropeptides mainly binds to µ and δ opioid receptors, while high-frequency stimulation of opioid peptides mainly binds to κ opioid receptors.

Zusanli (ST36), Yangming Stomach Meridian, is also one of the lower acupoints. Clinically, Zusanli (ST36) is the most commonly used acupoint for the treatment of all gastrointestinal diseases, and has a certain effect on preventing perioperative nausea and vomiting [25–26]. Duan [27] treated patients with gynecological laparoscopic surgery with single-point (Neiguan or Zusanli) and double-point TEAS respectively. Both stimulation methods can reduce the incidence of PONV in patients and relieve postoperative pain, but the effect of preventing PONV is basically the same. An animal study [28] explored the effect of electroacupuncture on intestinal motility in mice with postoperative intestinal paralysis. In the study, Zusanli single acupoint or combined with other abdominal acupoints was used. It was observed that electroacupuncture at Zusanli or combined with Tianshu could enhance postoperative gastrointestinal motility in mice and reduce the expression of inflammatory factors TNF-α and IL-6 (this may be one of the mechanisms). Zusanli (ST36), a key acupoint on the ‘Foot Yangming Stomach Meridian,’ traditionally used to “regulate the function of spleen and stomach,” exerts its influence through well-defined neurophysiological pathways. Modern research demonstrates that ST36 stimulation significantly modulates the autonomic nervous system, particularly by enhancing the activity of the vagal nerve [29]. This activation promotes gastric motility and helps normalize gastrointestinal function, providing a scientific basis for its efficacy in treating disorders like nausea and vomiting [30]. Furthermore, the traditional concept that ST36 can “adjust the balance of the triple energizer” finds a plausible explanation in contemporary neuroimmunology. The “Triple Energizer” (Sanjiao), while not a discrete anatomical organ, is increasingly understood as a functional concept representing the body-wide neuro-endocrine-immune (NEI) network that maintains homeostasis [31]. Stimulation at ST36 has been shown to activate the cholinergic anti-inflammatory pathway, a critical component of this network, thereby downregulating systemic pro-inflammatory cytokines like TNF-α and IL-6 [32]. By regulating this complex communication system, ST36 helps restore physiological balance, which aligns with the traditional action of “unblocking the qi movement.” The acupoints of Zusanli and Neiguan are compatible with each other, and the acupoints complement each other, which can enhance the therapeutic effect.

A key finding of this study was the significantly shorter time to first flatus in the combination group, which is a direct clinical indicator of accelerated gastrointestinal functional recovery. This outcome can be mechanistically linked to the well-documented influence of ST36 stimulation on vagal nerve activity. Research indicates that electroacupuncture at ST36 enhances gastrointestinal motility primarily by activating the vagal-cholinergic pathway, which involves the release of acetylcholine to promote intestinal peristalsis [33]. A prospective randomized controlled trial confirmed that stimulating ST36 in patients undergoing colorectal surgery significantly shortened the time to first flatus and defecation, an effect attributed to the excitation of vagal efferent fibers [34]. Therefore, the improvement observed in our study is likely a direct result of this neurogenic pathway, whereby the combined stimulation of PC6 and ST36 provided a potent activation of parasympathetic output to the gut, thereby more rapidly overcoming postoperative ileus. Furthermore, the superior efficacy of the combined stimulation in reducing both the incidence and severity of PONV integrates several complementary neurophysiological pathways. The antiemetic effect of PC6 is closely associated with the modulation of the central vomiting circuit. Specifically, stimulating PC6 modulates the vagus nerve-nucleus tractus solitarius pathway, where it regulates serotonin (5 HT3) related signaling, a cornerstone in the pathophysiology of nausea and vomiting [35]. This central action is complemented by the peripheral effects of ST36, which has been shown to downregulate the expression of 5 HT3 receptors in the colon, thereby reducing afferent emetic signals from the gastrointestinal tract [36]. This dual modulation of central and peripheral 5 HT3 pathways offers a comprehensive antiemetic strategy. Concurrently, stimulation at these acupoints, particularly PC6, triggers the release of beta endorphins [36], which not only contributes to analgesia but may also create a sense of well-being, indirectly alleviating the sensation of nausea. This multi-faceted mechanism, involving neurotransmitter modulation and endogenous opioid release, provides a plausible explanation for the robust antiemetic effects observed in our study. Beyond the direct gastrointestinal and antiemetic effects, the accelerated recovery observed may also be partly attributed to the anti-inflammatory properties of acupoint stimulation, particularly at ST36. Surgical trauma itself induces a systemic inflammatory response, characterized by the release of pro-inflammatory cytokines such as TNF-α and IL-6, which can delay the recovery process. Animal studies provide substantial evidence that electroacupuncture at ST36 can significantly reduce the expression of these key pro-inflammatory cytokines through neuro-immune modulation [37]. By mitigating the postoperative inflammatory cascade, ST36 stimulation may create a more favorable physiological environment for healing, thereby contributing to the overall improved recovery seen in our patients.

While our study was not designed to elucidate the underlying neurophysiological mechanisms, the superior clinical outcomes observed in the combination group (A) compared to the single-acupoint group (B) and the sham group (C) suggest an additive or synergistic clinical effect of co-stimulating PC6 and ST36. The significantly lower VAS scores and shorter time to first flatus in Group A particularly point towards an enhanced benefit on gastrointestinal function recovery that warrants further investigation. This finding is consistent with the clinical practice in traditional Chinese medicine of combining these acupoints to strengthen therapeutic efficacy [38]. The precise mechanisms behind this enhanced effect, such as whether it results from the concurrent modulation of divergent neural pathways as suggested by basic research [23, 24] or other integrated physiological responses, remain a fruitful area for future mechanistic studies employing techniques such as functional magnetic resonance imaging (fMRI) or measurement of specific neurotransmitters.

Furthermore, the statistically significant reductions in PONV and the accelerated gastrointestinal recovery observed in this study carry substantial clinical relevance. The dramatic decrease in PONV incidence in the combination group (Group A) to 6.7% (2/30) within the critical 0–6 h period, compared to 43.3% (13/30) in the sham control group (Group C), translates to a profound improvement in the immediate postoperative experience for patients. This represents an absolute risk reduction (ARR) of 36.6% and a number needed to treat (NNT) of approximately 3. This means that for every three patients treated with the combined acupoint stimulation, one additional case of early postoperative nausea is prevented. Preventing PONV directly enhances patient comfort and satisfaction, reduces the risk of complications such as wound strain or dehydration, and can decrease the nursing workload and the need for rescue antiemetic medications, thereby potentially lowering direct drug costs and indirect healthcare resource utilization. Similarly, the significantly shorter time to first flatus in Group A (22.03 ± 1.63 h) compared to Group C (26.57 ± 7.54 h) is not merely a statistical finding but a clear marker of enhanced recovery of gastrointestinal function. A quicker return of bowel function allows for earlier initiation of oral intake, which can prevent electrolyte imbalances, promote overall strength, and may contribute to a shorter length of hospital stay, particularly in settings where same-day discharge is targeted. These outcomes collectively demonstrate that the non-pharmacological intervention of combined acupoint stimulation offers a clinically meaningful benefit, aligning with the goals of enhanced recovery after surgery (ERAS) protocols by improving patient-centered outcomes and potentially optimizing the efficiency of postoperative care.

In this study, the synergistic anti-vomiting effect of combined stimulation of Neiguan (PC6) and Zusanli (ST36) was verified for the first time in hysteroscopic surgery, and the non-invasive shallow acupuncture pressing technique was innovatively adopted. For the first time, the first anal exhaust time after operation was used as a quantitative index to confirm that it significantly reduced postoperative nausea and vomiting and accelerated the recovery of gastrointestinal function, providing an efficient and safe non-drug intervention for the management of integrated traditional Chinese and Western medicine. Of course, this study also has some limitations. First of all, the included patients were all from a hospital, with regional and personnel restrictions. There are problems such as small sample size, short research time, and limited personnel. It is necessary to increase the number and scope of samples, extend the research time, and conduct deeper observations. Secondly, due to financial and time constraints, no animal experiments related to the research were carried out. Therefore, it is impossible to carry out relevant research on the mechanism of action, which needs to be further explored. Another limitation pertains to the blinding of personnel. In addition, the evaluation of patient blinding was performed only in a randomly selected subset of participants rather than in the full study population. Although the questionnaire results and Bang blinding index suggested that patient blinding was generally maintained, the limited sample used for blinding assessment may reduce the precision of this evaluation. Future trials should assess blinding in all enrolled participants and report standardized blinding indices as part of the trial quality assessment. While stringent measures were taken to blind patients and outcome assessors, the acupuncturists administering the real and sham interventions were necessarily aware of the group assignments. This could theoretically introduce performance bias. However, we mitigated this risk by standardizing all other aspects of patient care and anesthesia, and by using a credible sham device that ensured patient blinding, as confirmed by our blinding questionnaire. Furthermore, the primary outcomes (PONV incidence, VAS scores) were objective or patient-reported and were collected by blinded nursing staff, which reduces the likelihood that the acupuncturist’s knowledge directly influenced the results.

## Conclusions

In conclusion, combined with press needle stimulation at Zusanli and Neiguan can effectively reduce the severity of nausea and vomiting after gynecological hysteroscopic surgery, shorten the first anal exhaust after surgery, and help patients recover after surgery.

## Data Availability

All data generated or analyzed during this study are included in this published article.
